# Reference gene validation for qPCR in rat carotid body during postnatal development

**DOI:** 10.1186/1756-0500-4-440

**Published:** 2011-10-24

**Authors:** Insook Kim, Dongjin Yang, Xinyu Tang, John L Carroll

**Affiliations:** 1 University of Arkansas for Medical Sciences, Department of Pediatrics, Division of Pulmonary Medicine, Arkansas Children's Hospital Research Institute, Little Rock, Arkansas, USA; 2 University of Arkansas for Medical Sciences, Department of Pediatrics, Division of Biostatistics, Arkansas Children's Hospital Research Institute, Little Rock, Arkansas, USA

## Abstract

**Background:**

The carotid bodies are the main arterial oxygen chemoreceptors in mammals. Afferent neural output from the carotid bodies to brainstem respiratory and cardiovascular nuclei provides tonic input and mediates important protective responses to acute and chronic hypoxia. It is widely accepted that the selection of reference genes for mRNA normalization in quantitative real-time PCR must be validated for a given tissue and set of conditions. This is particularly important for studies in carotid body during early postnatal maturation as the arterial oxygen tension undergoes major changes from fetal to postnatal life, which may affect reference gene expression. In order to determine the most stable and suitable reference genes for the study of rat carotid body during development, six commonly used reference genes, β-actin, RPII (RNA polymerase II), PPIA (peptidyl-proyl-isomerase A), TBP (TATA-box binding protein), GAPDH, and 18s rRNA, were evaluated in two age groups (P0-1 and P14-16) under three environmental oxygen conditions (normoxia, chronic hypoxia and chronic hyperoxia) using the three most commonly used software programs, geNorm, NormFinder and BestKeeper.

**Findings:**

The three programs produced similar results but the reference gene rankings were not identical between programs or experimental conditions. Overall, 18s rRNA was the least stable reference gene for carotid body and, when hyperoxia and/or hypoxia conditions were included, actin was similarly unstable.

**Conclusions:**

Reference or housekeeping gene expression for qPCR studies of carotid body during postnatal development may vary with developmental stage and environmental conditions. Selection of the best reference gene or combination of reference genes for carotid body development studies should take environmental conditions into account. Two commonly used reference genes, 18s rRNA and actin, may be unsuitable for studies of carotid body maturation, especially if the study design includes altered oxygen conditions.

## Findings

### Introduction

Mammals have evolved a complex oxygen sensing system that links rapidly responding peripheral arterial PO_2 _sensors, the carotid body (CB) chemoreceptors, with central respiratory motor output and cardiovascular reflexes. Afferent neural output from the carotid bodies is transmitted via the carotid sinus nerves (CSN) to brainstem respiratory and cardiovascular nuclei, which control ventilatory, heart rate and blood pressure responses to hypoxia [1] and mediate other important defenses during hypoxic stress [2-6]. In all species studied to date, CB responsiveness to hypoxia is low in newborns and increases with age [7,8].

Glomus cells (or "type-1 cells"), the O_2_-sensing cells of the CB, are arranged in clusters with apposed nerve terminals of the carotid sinus nerve, whose cell bodies are located in the petrosal ganglion (PG) [9]. Studies of rat glomus cells from our laboratory demonstrate that the magnitude of hypoxia-induced cell membrane depolarization, the intracellular calcium response ([Ca^2+^]_i_) to hypoxia and the magnitude of an O_2_-sensitive background K^+ ^current increase with age from birth (postnatal day 0: P0) to postnatal day 14-21 (P14-21) [10-12]. The mechanisms are unknown but may involve postnatal changes in glomus cell ion channel expression. Therefore, studies of ion channel expression are crucial to understanding the mechanisms of CB glomus cell O_2 _sensing and its postnatal maturation.

Developmental studies of gene expression typically employ relative quantification by qPCR, where normalization against an internal control gene is commonly used. The use of internal control genes (reference genes) assumes that their expression is invariant in the cells or tissue under study and with experimental treatments. Developmental studies, in addition, assume that reference genes for qPCR do not change with stage of development or ambient conditions. However, numerous studies show that commonly used reference genes, such as β-actin, GAPDH or 18s rRNA, are not constant between different developmental stages and different experimental conditions [13-20]. Normalization of the data with unstable reference genes can result in misleading or false conclusions.

It has become widely accepted that the selection of reference genes must be validated for a given tissue and set of conditions [21] and the use of multiple reference genes is viewed as a more robust, accurate and reliable approach to normalization [21-24]. This approach can be facilitated by the use of tools such as geNorm [22], NormFinder [25] and BestKeeper [26], which allow selection of the most stable reference genes and determination of the best combination to use for normalization for a given tissue and set of conditions.

Fetal arterial PO_2 _in mammals is about 23-28 mmHg and rises ~ 4 fold within the first two hours after birth [27,28], raising the additional possibility that oxygen-dependent gene expression may change during the first days or weeks after birth [29,30]. Specifically, oxygen tension may affect the expression of reference genes in a tissue-specific manner [13,31], suggesting that candidate reference genes for normalization should be validated not only for a given tissue but also for oxygen conditions that may affect expression and over the time frame when such changes may occur.

In the present study, six candidate reference genes were evaluated using three reference gene selection tools in whole rat carotid bodies during a crucial developmental period (P0 to P16) and under conditions of peri- and postnatal normoxia, hypoxia and hyperoxia. Results indicate that actin and 18s rRNA, housekeeping genes commonly used for qPCR normalization, were among the least stable reference genes under most conditions.

## Methods

### Overview of study design

Six commonly used reference genes, β-actin, RPII (RNA polymerase II), PPIA (peptidyl-proyl-isomerase A), TBP (TATA-box binding protein), GAPDH, and 18s rRNA, were validated using geNorm [22], NormFinder [25] and BestKeeper [26] for two age groups (P0-1 and P14-16) under three environmental oxygen conditions (normoxia, chronic hypoxia and chronic hyperoxia). This yielded four groups as follows: age P0-1, normoxia (N1), age P14-16, normoxia (N14), age P14-16, chronic hyperoxia treated (Hyper14), and age P14-16, chronic hypoxia treated (Hypo14). To reduce the inter-assay variations, all six reference genes were run with qPCR with all four sets of samples; N1, N14, Hyper14, and Hypo14. In order to evaluate CB reference gene stability under conditions relevant to postnatal development we performed the analysis, with geNorm, NormFinder and BestKeeper, using four combinations of the conditions, as follows: N1+N14 (development), N14+Hyper14 (chronic hyperoxia during development), N14+Hypo14 (chronic hypoxia during development) and N1+N14+Hyper14+Hypo14 (development + altered environmental condition). The procedures used in this study were approved by the Institutional Animal Care and Use Committee of the University of Arkansas for Medical Sciences.

### Chronic Hyperoxia or Hypoxia Treatment

For chronic hyperoxia or hypoxia treatment, timed-pregnant Sprague-Dawley rats were placed in a controlled atmosphere chamber 1-2 days prior to expected delivery and were allowed to give birth in the chamber (OxyCycler Model A84XOV, BioSpherix, Redfield, NY). The system continuously monitors and maintains a preset oxygen level, and O_2_/CO_2 _tensions are recorded continuously (AnaWin software). The chambers employ a controlled leak to the room environment in order to limit CO_2_/humidity buildup. For chronic hyperoxia and hypoxia treatment, rats were exposed to 0.60 FiO_2 _or 0.12 FiO_2_, respectively. Pups and dams were maintained in the chamber until use at P14-16.

### Isolation of Rat CB and tRNA Extraction

Carotid bodies (CB) were isolated from rats age P0-1 (N1), P14-16 (N14), P14-16 male and female rats exposed continuously to hyperoxia (60% O_2_) from birth (Hyper14), and P14-16 rats exposed to hypoxia (12% O_2_) from birth (Hypo14) as described previously [12]. For CB isolation, rats were anesthetized with isoflurane and decapitated under deep surgical anesthesia. The carotid bifurcations were dissected and placed in ice-cold phosphate buffered saline (PBS). The carotid bodies were then removed from the bifurcations and placed in cold sterile PBS. Isolated CBs were placed into a 1.5 ml centrifuge tube, washed with ice-cold PBS, and stored in RNAlater stabilization reagent (Qiagen) at -80°F. Frozen collected CBs were processed to extract the Total RNA (tRNA) using AquaPure RNA isolation kit (Bio-Rad). tRNA was extracted from ~ 50 frozen CBs and the tRNA pellet was reconstituted in 10-16 μl of hydration solution depending on size of isolated CB. The concentration of extracted tRNA was measured by spectrophotometer (SmartSpec Plus, Bio-Rad) at 260 nm. Purity of tRNA was determined as the 260 nm/280 nm ratio with expected values between 1.8 and 2.

Extracted tRNA were treated with RQ1 RNA-free DNase (Promega) and estimated 1 μg of pure tRNA ran for cDNA sysnthesis (20 μl) by using iScript cDNA synthesis kit (Bio-Rad). cDNA at 5X dilution were run using qPCR in triplicate. The six putative reference genes, β-actin, RPII (RNA polymerase II), PPIA (peptidyl-proyl-isomerase A), TBP (TATA-box binding protein), GAPDH, and 18s rRNA, were evaluated using qPCR. All six reference genes were tested with cDNA synthesized from N1, N14, Hyper14, and Hypo14 on the same plate to reduce inter-assay variations.

### Quantitative Real Time RT-PCR (qPCR)

Primers for reference genes were designed by using Beacon Designer 2.0; PPIA, TBP, RPII, β-actin, GAPDH, 18s rRNA, TASK-1, TASK-2 and TASK-3 (Table 1). Designed primers were ordered from Integrated DNA Technologies (IDT, Coraville, IA) and tested on cDNA from positive control tissues prior to testing on cDNA of CB. The PCR efficiency of each primer pair was tested with the serial dilution of cDNA prepared with N14 CB in triplicate (Table 1). The real-time PCR efficiency rate (E) in the exponential phase was calculated according to the following equation [32]:

**Table 1:** Information on the primers used for real time PCR

Gene	Accession #		Primers	Product Size (bp)	Efficiency Rate (E)
PPIA	NM_017101	F	GTCAACCCCACCGTGTTCTTC	133	1.93
		R	ATCCTTTCTCCCCAGTGCTCAG		
TBP	NM_001004198	F	ACCGTGAATCTTGGCTGTAAAC	123	2.06
		R	CGCAGTTGTTCGTGGCTCTC		
RPII	AB017711	F	GGCTCTCCAGATTGCGATGTG	124	1.93
		R	CAGGTAACGGCGAATGATGATG		
β-actin	NM_031144	F	CAGGGTGTGATGGTGGGTATGG	115	2.03
		R	AGTTGGTGACAATGCCGTGTTC		
GAPDH	NM_017008	F	CAAGTTCAACGGCACAGTCAAG	123	1.91
		R	ACATACTCAGCACCAGCATCAC		
18s	X01117	F	CACGGGTGACGGGGAATCAG	105	2.03
		R	CGGGTCGGGAGTGGGTAATTTG		
TASK-1	NM_033376	F	GCAGAAGCCGCAGGAGTTG	126	2
		R	GCCCGCACAGTTGGAGATTTAG		
TASK-2	AM229406	F	ACGCCCTCTACCGCTACTTTG	129	2.12
		R	GCCGCCTCCTCCTCTTCTTG		
TASK-3	NM_053405	F	CGGTGCCTTCCTCAATCTTGTG	144	1.8
		R	TGGTGCCTCTTGCGACTCTG		

Nucleotide sequences for the primers, size of PCR products, and PCR amplification efficiency rate of each primer set. All the genes are from rat origin. The real-time PCR efficiency rate (E) in exponential phase was calculated according to the equation: E = 10[-1/slope] [32].

E=10[-1∕slope]-1

To exclude genomic DNA contamination, tRNA was treated with RQ1 RNase-free DNase (Promega) as described above (tRNA extraction methods) and cDNA was synthesized using iScript cDNA synthesis kit (Bio-Rad). To check for DNA contamination on newly made tRNA, cDNA without reverse transcriptase (-RT) was synthesized and tested prior to use. To prevent false detection with the qPCR, a no-template-control (NTC) was tested on every run for each primer set and the melting curve was checked.

Real time PCR using SYBR green technology was performed on an iCycler iQ real-time detection system in 96-well microtitre plates using a final volume of 20 μl (Bio-Rad). SYBR Green Supermix (Bio-Rad and Applied Biosystems) and 0.1 μM of primers were mixed with DNAse and RNase-free water to make the 9/10^th ^of the total reaction volume and 1/10^th ^of cDNA was added into the mixture. The following amplification program was used: after 5 min of denaturation at 95°C, 50 cycles of real time PCR with 2-step amplification were performed consisting of 15s at 95°C for denaturation, 45s at 60°C for annealing and 1min at 95°C for polymerase elongation. In each qPCR run, all six reference genes were run on all four conditions, N1, N14, Hyper 14, and Hypo 14, in one 96 well PCR plate simultaneously. All samples were amplified in triplicate and the mean was obtained for further calculation. C_T _values of 50 were excluded from further mathematical calculation, because 50 represents no quantitative information of the RNA amount, but only the end of the PCR run.

### Data analysis

#### Reference gene ranking and selection of best combination of multiple reference genes

Three popular software programs, geNorm [22], NormFinder [25] and BestKeeper [26], were used to evaluate or validate the best reference genes for use during CB development. In general, they are based on the principle that the expression of reference genes should be the same under all experimental conditions and cell types studied. geNorm measures the variation between any two candidate control genes as the standard deviation of (log-transformed) control gene ratios. Each candidate reference gene is assigned a stability measure (M) by comparing it (in a pairwise fashion) with each other reference gene candidate. The candidate genes with the most stable expression have the lowest M. The genes are then ranked, using stepwise exclusion of the least stable genes. NormFinder [25] uses a model-based approach to estimate overall reference gene stability but also considers variations between sample subgroups. BestKeeper [26] uses repeated pair-wise correlation analysis to determine the optimal reference genes. For relative gene quantification, REST 384 or REST2009 were used [33].

#### Statistical Method for Rank Aggregation

These three programs, geNorm, NormFinder and BestKeeper, have been previously reported to yield different rankings of reference genes [34]. As they use different approaches to evaluating reference gene stability [34], they would not be expected to yield identical results. Therefore, weighted rank aggregation was performed to combine the ordered lists of genes produced by geNorm, NormFinder, and BestKeeper to a consensus rank of genes. The M-values obtained from geNorm, variability measurements from NormFinder, and the coefficients of correlation from BestKeeper were used as weights in the aggregation process. Brute force method was used to enumerate all possible candidate lists and find the one with the minimum Spearman footrule distance using the BruteAggreg function [35]. Although it is recommended that the Cross-Entropy Monte Carlo algorithm should be used when the size of the ranking list is relatively larger than 10, it was used additionally to validate the consensus rank of genes resulting from the brute force approach. The two methods yielded consistent ranking lists, demonstrating that the consensus ranks of genes were robust to the methods used. The rank aggregations were conducted with R software version 2.11.1 (R Development Core Team, 2009).

## Results

**Figure 1:**
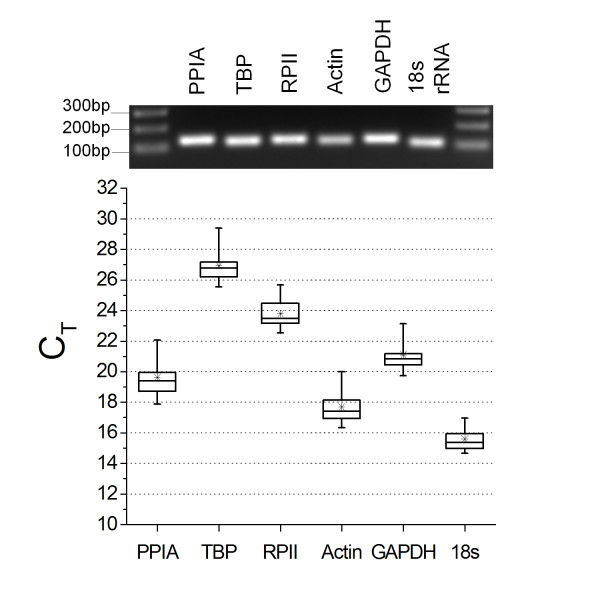
**The distribution of gene expression levels of tested reference genes in pooled C_T _values**. Data obtained from immature (N1), mature (N14), chronic hyperoxia (Hyper14), and chronic hypoxia (Hypo14) treated rat CB. Top panel: PCR amplicons from all tested reference genes were detected on 1.5% agarose gel as single band and correct size of PCR product. Bottom panel: C_T _values for each reference gene are shown as medians (line in box), means (asterisks), 25 to 75 percentile (boxes), and 1 to 99 percentile ranges (whiskers).

All six reference genes, PPIA, TBP, RPII, β-actin, GAPDH, and 18s rRNA, were detected from all tested CB samples (Figure 1). The median expression levels (C_T _value) for each validated reference gene are shown in Figure 1. For 18s rRNA, cDNA was diluted 125x to fit its C_T _value into a reasonable C_T _range. TBP showed the lowest expression level among 6 reference genes, but its C_T _values were at a reasonable detection level for SYBR green real time PCR.

**Figure 2:**
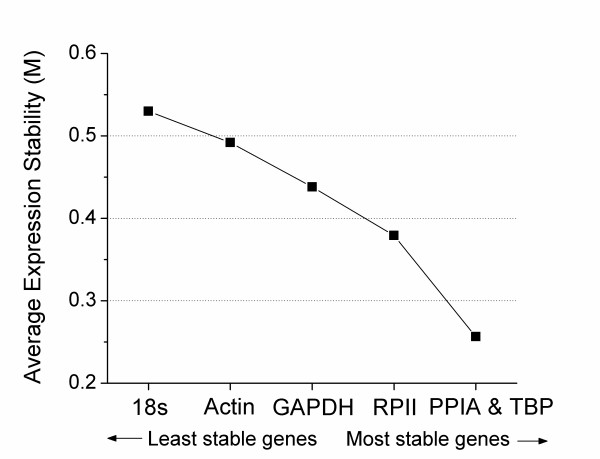
**geNorm analysis of the 6 candidate reference genes in all pooled data from N1, N14, Hyper14, and Hypo14 rat CB (n = 28)**. Average expression stability of candidate reference gene was determined by stepwise exclusion of least stable genes. Lower M values indicate greater stability.

Reference genes during CB development in normoxia, chronic hyperoxia and chronic hypoxia treatment were evaluated with extracted RNA in four combinations as follows: N1+N14, N14+Hyper14, N14+Hypo14, or N1+N14+Hyper14+Hypo14. geNorm found PPIA and TBP or RPII and TBP to be the most stable reference gene combinations (Table 2). 18s rRNA was the least stable in all groups according to geNorm (Table 2). geNorm calculates an expression stability value, termed "M", which is highest for the least stable gene and lowest for the most stable (Figure 2). The PPIA and TBP combination was by far the most stable and 18s rRNA was the least stable reference gene (Figure 2).

**Table 2:** Ranking of the 6 selected reference genes in rat whole carotid bodies by geNorm, NormFinder and BestKeeper

geNorm	N1+N14 (n = 16)		N14+Hyper14 (n = 14)		N14+Hypo14 (n = 14)		N1+N14+Hyper14+ Hypo14 (n = 28)	
**Rank**	**Gene**	**M-value**	**Gene**	**M-value**	**Gene**	**M-value**	**Gene**	**M-value**

Best two genes	RPII TBP	0.26	PPIA TBP	0.20	PPIA TBP	0.29	PPIA TBP	0.26

3	PPIA	0.27	RPII	0.35	RPII	0.36	RPII	0.38
4	actin	0.34	GAPDH	0.43	GAPDH	0.45	GAPDH	0.44
5	GAPDH	0.38	actin	0.50	actin	0.50	actin	0.49
6	18s	0.40	18s	0.57	18s	0.52	18s	0.53

**Norm Finder**	N1+N14 (n = 16)		N14+Hyper14 (n = 14)		N14+Hypo14 (n = 14)		N1+ N14+Hyper14+ Hypo14 (n = 28)	

Rank	Gene	Variability	Gene	Variability	Gene	Variability	Gene	Variability

Best two genes	TBP Actin	0.009	RPII GAPDH	0.007	RPII GAPDH	0.008	PPIA TBP	0.011

1	GAPDH	0.01	RPII	0.013	TBP	0.007	TBP	0.015
2	PPIA	0.011	GAPDH	0.013	PPIA	0.015	GAPDH	0.017
3	actin	0.014	TBP	0.014	GAPDH	0.018	18s	0.019
4	TBP	0.015	actin	0.019	RPII	0.018	PPIA	0.020
5	RPII	0.016	PPIA	0.021	18s	0.020	RPII	0.021
6	18s	0.018	18s	0.021	actin	0.021	actin	0.023

**Best Keeper**	N1+N14 (n = 16)		N14+ yper14 (n = 14)		N14+Hypo14 (n = 14)		N1 + N14 + Hyper14+ Hypo14 (n = 28)	

Rank	Gene	Coeff. of corr.[r]	Gene	Coeff. of corr.[r]	Gene	Coeff. of corr.[r]	Gene	Coeff. of corr.[r]

1	PPIA	0.89	RPII	0.97	RPII	0.86	PPIA	0.97
2	actin	0.84	PPIA	0.96	PPIA	0.85	TBP	0.95
3	GAPDH	0.83	GAPDH	0.96	TBP	0.85	GAPDH	0.95
4	18s	0.73	TBP	0.95	actin	0.75	RPII	0.93
5	RPII	0.72	actin	0.93	GAPDH	0.69	actin	0.92
6	TBP	0.70	18s	0.87	18s	0.56	18s	0.89

The reference genes are ranked in several groups of CB as follows: N1+N14 = immature and mature CB, reared in normoxia (n = 16). N14+Hyper14 = mature normoxia and hyperoxia treated CB (n = 14). N14+Hypo14 = mature normoxia and hypoxia treated CB (n = 14). N1+N14+Hyper14+Hypo14 = immature, normoxia (N1), mature, normoxia (N14), mature, hyperoxia (Hyper14), and mature, hypoxia treated (Hypo14) CB (n = 28). Genes are ranked on their stability value calculated by each respective program: geNorm, M-value; NormFinder, Variability; and BestKeeper, Coefficient of correlation [r]. (n) = number of independent determinations.

For the combination of all four conditions, N1+N14+Hyper14+Hypo14, NormFinder also selected PPIA and TBP as the best two reference genes (Table 2) and BestKeeper ranked PPIA and TBP as the top two most stable reference genes (Table 2). Rankings for other combinations of conditions varied more with NormFinder and BestKeeper, compared to geNorm (Table 2). Similar to the 18s rankings produced by geNorm, 18s rRNA tended to rank near the bottom of stability rankings using NormFinder and BestKeeper (Table 2).

As the three programs produced different results, we attempted to determine consensus rankings using weighted rank aggregation. The most consistent result of consensus rankings was that 18s rRNA ranked at or near the bottom of the rankings under all conditions (Table 3). For development alone (N1+N14) PPIA and GAPDH ranked as the top two reference genes by consensus analysis (Table 3). For hyperoxia during development (N14+Hyper14) RPII and TBP were ranked as the top two genes by consensus (Table 3). In both groups involving hypoxia during development (N14+Hypo14 and N1+N14+Hyper14+Hypo14) TBP and PPIA were ranked, by consensus, as the top two most stable reference genes (Table 3). When the group included hyperoxia or hypoxia during development, actin ranked at or near the bottom of stability rankings (Table 3).

**Table 3:** Consensus ranking

Gene ranking for group N1+N14 (n = 16)				
**Rank Position**	**geNorm**	**NormFinder**	**BestKeeper**	**Consensus**

1	RPII	GAPDH	PPIA	PPIA
2	TBP	PPIA	Actin	GAPDH
3	PPIA	Actin	GAPDH	Actin
4	Actin	TBP	18s	RPII
5	GAPDH	RPII	RPII	TBP
6	18s	18s	TBP	18s

Gene ranking for group N14+Hyper14 (n = 14)				

Rank Position	geNorm	NormFinder	BestKeeper	Consensus

1	PPIA	RPII	RPII	RPII
2	TBP	GAPDH	PPIA	TBP
3	RPII	TBP	GAPDH	GAPDH
4	GAPDH	Actin	TBP	PPIA
5	Actin	PPIA	Actin	Actin
6	18s	18s	18s	18s

Gene ranking for group N14+Hypo14 (n = 14)				

Rank Position	geNorm	NormFinder	BestKeeper	Consensus
1	PPIA	TBP	RPII	TBP
2	TBP	PPIA	PPIA	PPIA
3	RPII	GAPDH	TBP	RPII
4	GAPDH	RPII	Actin	GAPDH
5	Actin	18s	GAPDH	Actin
6	18s	Actin	18s	18s

Gene ranking for group N1+N14+Hyper14+Hypo14 (n = 28)				

Rank Position	geNorm	NormFinder	BestKeeper	Consensus
1	PPIA	TBP	PPIA	TBP
2	TBP	GAPDH	TBP	PPIA
3	RPII	18s	GAPDH	GAPDH
4	GAPDH	PPIA	RPII	RPII
5	Actin	RPII	Actin	18s
6	18s	Actin	18s	Actin
				

Weighted rank aggregation was performed to combine the ordered lists of genes produced by geNorm, NormFinder, and BestKeeper to a consensus rank of genes.

Another potential source of variability is the source of the qPCR master mixture. Therefore, the six reference genes were tested with iQ SYBR green supermix from Bio-Rad and compared with PCR master mix from Applied Biosystems, using geNorm, NormFinder and BestKeeper (Table 4). Overall, the Applied Biosystems SYBR green mastermix provided more consistent rankings across the three software programs. However, in spite of the greater variability in rankings in the Bio-Rad group, the consensus rankings were nearly identical for the two different vendors (Table 4).

**Table 4:** Gene ranking and consensus ranking using SYBR green super mixtures from two different vendors, Bio-Rad and Applied Biosystems

	Gene and consensus ranking for group N1+N14 (n = 16) using Bio-Rad SYBR						
	
	Bio-Rad SYBR						
	
Rank Position	geNorm	M-value	NormFinder	Variability	BestKeeper	Coeff. of corr. [r]	Consensus
1	TBP	0.26	GAPDH	0.01	PPIA	0.89	PPIA
2	RPII	0.26	PPIA	0.011	Actin	0.84	GAPDH
3	PPIA	0.27	Actin	0.014	GAPDH	0.83	Actin
4	Actin	0.34	TBP	0.015	18s	0.73	TBP
5	GAPDH	0.38	RPII	0.016	RPII	0.72	RPII
6	18s	0.40	18s	0.018	TBP	0.70	18s

							
	Gene and consensus ranking for group N1 + N14 (n = 16) using Applied Biosystems SYBR						
	
	Applied Biosystems SYBR						
	
Rank Position	geNorm	M-value	NormFinder	Variability	BestKeeper	Coeff. of corr. [r]	Consensus

1	PPIA	0.21	PPIA	0.008	PPIA	0.99	PPIA
2	GAPDH	0.21	GAPDH	0.013	GAPDH	0.99	GAPDH
3	Actin	0.25	Actin	0.013	Actin	0.99	Actin
4	RPII	0.32	RPII	0.014	RPII	0.98	RPII
5	TBP	0.34	TBP	0.016	TBP	0.98	TBP
6	18s	0.39	18s	0.024	18s	0.97	18s

The reference genes are ranked for N1+N14 group by geNorm, Normfinder and BestKeeper and the consensus ranking determined by rank aggregation. (n) = number of independent determinations.

The impact of reference gene choice was evaluated by determining the relative gene expression ratios of three relevant targets during CB development, genes for TASK-1, TASK-2 and TASK-3 potassium channels. Their relative expression during development was evaluated with REST2009 (http://www.gene-quantification.de/rest-2009.html) using the stable combination PPIA and TBP, as determined by geNorm, vs. the least stable reference gene, 18s rRNA. As shown in table 5, TASK-2 expression was found to be significantly down-regulated during development when PPIA+TBP were the reference genes, but not when 18s rRNA was used as a reference gene.

**Table 5:** Relative gene expression ratios of three interest TASK channel genes, TASK-1, TASK-2, and TASK-3, were compared by using one least stable reference gene (18s rRNA) and two best stable reference genes (PPIA and TBP)

Reference Gene	TASK-1 (*p *value, n)	TASK-2 (*p *value, n)	TASK-3 (*p *value, n)
18s rRNA	1.172 (0.756, n = 11)	0.682 (0.442, n = 8)	1.285 (0.661, n = 12)

PPIA & TBP	0.69 (0.183, n = 13)	0.29 (0.000, n = 11) Down	0.925 (0.545, n = 13)

The ratios and statistical *p *values of three interest genes in groups N1+N14 were analyzed by REST2009 software. (n) = number of independent determinations.

## Discussion

We investigated the expression stability in rat whole carotid body of multiple commonly used qPCR reference genes, during early postnatal development when rat CB O_2_-sensing maturation takes place [10-12,36,37], using three popular software programs for reference gene selection as well as qPCR reagents from different vendors. Although the three programs produced similar results, the rankings were not identical and, in some cases, were substantially different. With respect to agreement between the three programs, for the combination of all conditions (N1+N14+Hyper14+Hypo14) geNorm and NormFinder selected PPIA+TBP as the best combination of multiple reference genes and BestKeeper selected PPIA and TBP as the two highest ranked (more stable) reference genes. The results indicated that 18s rRNA was the least stable reference gene for CB overall and, when hyperoxia and/or hypoxia conditions are included, actin was similarly unstable. The use of reagents from different vendors may substantially impact reference gene stability rankings.

geNorm produced the most consistent results across all developmental/oxygen conditions, selecting PPIA+TBP as the best multiple reference gene combination in three of four groups (Table 2). The M-value for the multiple reference gene combination PRII+TBP, selected by geNorm for N1+N14, is very close to that for PPIA, suggesting that PPIA+TBP would be a good choice of reference genes for all groups (Table 2).

Although our study was not designed to measure the effect of altered oxygen environment on individual reference gene expression, it appears that hyperoxia and hypoxia affect the stability rankings of specific genes. For example, NormFinder ranked actin as one of the best two reference genes for N1+N14, while actin was ranked as the least stable reference gene for the two groups that included chronic hypoxia (Table 2). Similarly, BestKeeper ranked actin as one of the most stable reference genes for development (N1+N14), while actin was ranked among the least stable in the groups that included chronic hyperoxia or hypoxia (Table 2). Thus, the effect of hypoxia on housekeeping gene expression may vary with experimental conditions and should be tested for a given tissue and set of conditions. We did not investigate the effects of chronic intermittent hypoxia (CIH) on reference gene stability because there are many different CIH exposure paradigms and such a large undertaking was beyond the scope of the present study.

An obvious potential limitation of this study is that better combinations of reference genes may exist, for the study of rat CB development, beyond the ones chosen. The choice of the six candidate reference genes studied was based on their common use and evidence that they are stable so-called "housekeeping genes" in other tissues. Never-the-less, others may exist that will turn out to be equally or more stable and suitable for developmental carotid body studies.

Our results add to a growing body of literature showing that reference or housekeeping gene expression for qPCR may vary with developmental stage and environmental conditions, and the specific genes, pattern and timing of variation may be tissue-specific [38]. It is also important to consider that our results are likely to be species-specific and may be developmental time-frame specific; studies of carotid body maturation in other species should validate reference genes for each species, O_2 _conditions and developmental time-frame.

## Competing interests

Statement for all authors:

The authors declare that they have no competing interests.

## Authors' contributions

IK participated in study concept and design, carried out the qPCR studies and participated in drafting the manuscript. DY helped with the qPCR studies. XT performed rank aggregation statistical analysis and participated in writing the manuscript. JC participated in study concept, design and drafting the manuscript. All authors read and approved the final manuscript.
